# Clinical Features and Outcomes of Patients with Pancreaticobiliary Malignancies in Los Angeles County and Their Association with CA 19-9 Levels

**DOI:** 10.3390/cancers15061723

**Published:** 2023-03-11

**Authors:** Jade Law, Harry Trieu, Guneet Kaleka, Joanna Turkiewicz, Samantha Palmer, Jennifer M. Lee, Kathryn T. Chen, James H. Tabibian

**Affiliations:** 1 LAC-USC Hematology and Oncology Fellowship Program, Los Angeles, CA 90033, USA; 2 LAC-USC Internal Medicine Residency Program, Los Angeles, CA 90033, USA; 3 UCLA-Olive View Internal Medicine Residency Program, Sylmar, CA 91342, USA; 4 Harbor-UCLA Medical Center, Division of Hematology and Medical Oncology, Torrance, CA 90502, USA; 5 Harbor-UCLA Medical Center, Division of Surgical Oncology, Torrance, CA 90502, USA; 6 UCLA-Olive View, Division of Gastroenterology, Sylmar, CA 91342, USA; 7 UCLA Vatche and Tamar Manoukian Division of Digestive Diseases, David Geffen School of Medicine at UCLA, Los Angeles, CA 90095, USA

**Keywords:** early detection of cancer, biomarkers, adenocarcinoma, pancreatic carcinoma

## Abstract

**Simple Summary:**

Pancreaticobiliary malignancies (PBMs) are a leading cause of cancer-related deaths worldwide, with 611,722 deaths reported in 2020. Serum carbohydrate antigen (CA) 19-9 is a tumor marker commonly used clinically in the management of patients with PBMs. Several studies have demonstrated the utility of CA 19-9 levels as a marker of diagnosis, prognosis, and surveillance. However, few studies have specifically examined clinical presentations and outcomes in patients with low or undetectable CA 19-9 levels. The aim of our retrospective study was to examine the clinical features and outcomes of patients with tissue-proven cases of PBMs who had low CA 19-9 levels at diagnosis and compare them to those with normal and elevated CA 19-9 levels. Given the morbidity and mortality of PBMs, a better understanding of the role of CA 19-9 could lead to the tailored management of disease and, thus, improved outcomes in those with low CA 19-9 levels.

**Abstract:**

Although CA 19-9 is a commonly used tumor marker in the management of PBMs, the literature describing outcomes in patients with PBMs who have undetectable or low (hereinafter “low”) CA 19-9 levels remains scarce. In this study, we sought to compare clinical features and outcomes in patients with PBMs and low CA 19-9 levels to those with normal and elevated CA 19-9 levels. Methods: We retrospectively collected data on patients with biopsy-confirmed PBMs and stratified patients into categories based on their CA 19-9 level at diagnosis. Survival curves were estimated for patients in each of the three aforementioned CA 19-9 groups using the Kaplan–Meier method and compared using a Cox proportional hazards regression model. Results: Of the 283 patients identified, 23 (8.1%) had low, 70 (24.7%) had normal, and 190 (67.1%) had elevated CA 19-9 levels. After controlling for sex, age, BMI, the presence of metastases at the time of diagnosis, and treatment with curative intent, the hazard ratio for death in the elevated CA 19-9 group compared to the low CA 19-9 group was 1.993 (95% CI 1.089–3.648; *p* = 0.025). Conclusion: The elevated CA 19-9 level compared to the low CA 19-9 level and the presence of metastases were associated with an increased hazard of death, while treatment with curative intent was associated with a decreased hazard of death.

## 1. Introduction

Pancreaticobiliary malignancies (PBMs) are a leading cause of cancer-related deaths worldwide, with 550,698 new cases and 611,722 deaths reported in 2020 [1]. In the United States alone, there were 72,410 new cases and 52,530 deaths attributed to PBMs in 2021 [2]. Despite advancements in the management of PBMs, the 5-year relative survival rate is still estimated at approximately 10% based on the Surveillance, Epidemiology, and End Results (SEER) program data [3]. This poor overall prognosis of PBMs is due in part to the difficulty of making an early diagnosis, limited treatment options at more advanced stages, and aggressive tumor biology [4,5].

Serum carbohydrate antigen (CA) 19-9 is a tumor marker frequently used clinically in the management of patients with PBMs, including pancreatic adenocarcinoma (PDAC), cholangiocarcinoma (CCA), and gallbladder carcinoma (GBCA). CA 19-9 has a sensitivity of 79–81% and a specificity of 82–90% in patients with PBMs; however, a low positive predictive value of 72.3% renders it less useful as a screening test [6]. Despite its widespread use, CA 19-9 is not specific to PBMs for several reasons. First, CA 19-9 levels can be increased in other malignancies, such as ovarian mucinous carcinoma, endometrial cancer, and lung cancer [7,8,9]. Second, CA 19-9 levels can be elevated in several benign conditions as well, including biliary obstruction, pancreatitis, or renal failure [10,11,12,13,14,15]. Third, one of the main limitations of CA 19-9, a monosialylated Lewis A blood group antigen, is that most Lewis antigen-negative individuals produce CA 19-9 at lower levels or cannot produce CA 19-9 at all [16]. There are three common Lewis antigen phenotypes that can be formed with Lewis antigenic epitopes: Le^a^ and Le^b^: Le(^a−b−^), Le(^a+b−^), and Le(^a−b+^). Individuals who are Lewis-negative (Le^a−b−^) are unable to synthesize CA 19-9. As a result, these Lewis-negative patients may be incorrectly considered to have low or undetectable CA 19-9 tumor markers even when a PBM is present. Notably, 5–10% of the Caucasian population have a Lewis-negative phenotype and cannot produce the CA 19-9 tumor antigen [6,10].

Several studies have demonstrated the utility of CA 19-9 levels as a marker for diagnosis, prognosis, and surveillance. For example, elevated levels of CA 19-9 in the setting of known PDAC are associated with poor prognosis and recurrence following treatment. Lower levels of CA 19-9 after treatment when compared to pre-treatment CA 19-9 levels have been shown to signify a favorable prognosis [17,18,19,20,21,22,23,24,25]. Few studies, however, have specifically examined clinical presentations and outcomes in patients with low or undetectable (hereinafter denoted as “low”) CA 19-9 levels. Given the morbidity and mortality of PBMs, a better understanding of the role of CA 19-9 could lead to the tailored management of disease and, thus, improved outcomes in those with low CA 19-9 levels. 

In this study, we examined the clinical features and outcomes of patients in the Los Angeles County Department of Health Services (LADHS) with tissue-proven cases of PDAC, CCA, or GBCA who had low CA 19-9 levels at diagnosis and compared them to those with normal and elevated CA 19-9 levels. Of note, LADHS embodies the second-largest municipal healthcare system in the country and has an understudied population of mostly minority patients. 

## 2. Materials and Methods

### 2.1. Study Population

We retrospectively queried an LADHS-wide database of patient encounters at Olive View-UCLA Medical Center (OVMC) and Harbor-UCLA Medical Center (HUMC), both located in Los Angeles, CA, USA, for patients with a diagnosis matching ICD-10-CM codes for PBMs (Table 1). Electronic health records of these patients were further reviewed, and those with tissue-proven PBMs diagnosed between the years 2014 and 2020 were included in the study. Patients without a documented tissue diagnosis of PBMs or without a CA 19-9 level within 30 days of diagnosis were excluded.

### 2.2. Study Variables and Primary Outcome

Demographic and clinical data abstracted from health records included age, sex, ethnicity, body mass index (BMI), history of smoking, history of non-gastrointestinal (GI) cancer, history of comorbidities (diabetes mellitus, hypertension, congestive heart failure, coronary artery disease, chronic obstructive pulmonary disease, and stroke), and type of treatment received (curative or palliative). Laboratory data abstracted included CA 19-9, total bilirubin level, carcinoembryonic antigen level, and CA 125 level at the time of diagnosis [26,27]. In addition, we abstracted the cancer stage and Eastern Cooperative Oncology Group (ECOG) performance status at time of diagnosis. Overall survival was defined as time from date of diagnosis to the date of death or last known follow-up. This constituted the primary outcome of the study.

### 2.3. CA 19-9 Assays and Patient Stratification Based on CA 19-9 Level

During the study period (between 2014 and 2020), two different automated CA 19-9 laboratory assays were used: ADVIA Centaur (Bayer Diagnostics, with headquarters in Tarrytown, NY, USA) and Elecsys E170 (Roche Diagnostics, with North American headquarters in Indianapolis, IN, USA). At the study’s institutions, the limits of detection for these two assays were <2 or ≤3 U/mL; thus, for our analysis, patients were stratified into three groups according to the CA 19-9 level at the time of diagnosis: (1) low (≤3 U/mL), (2) normal (4–35 U/mL), and (3) elevated (over 35 U/mL). 

### 2.4. Statistical Analysis

Descriptive statistics were computed for patients with low, normal, and elevated CA 19-9 levels at diagnosis. Survival curves were estimated for patients in each of the three aforementioned CA 19-9 groups using the Kaplan–Meier method and compared using a Cox proportional hazards regression model. Median follow-up times were estimated using the reverse Kaplan–Meier estimator. A *p*-value < 0.05 was considered statistically significant. All statistical analyses were performed using Stata/IC 16.1 (StataCorp, College Station, TX, USA).

## 3. Results

### 3.1. Characteristics of the Study Sample

We identified a total of 421 patients with a diagnosis matching 1 of 12 ICD-10-CM codes for PBM (Table 1). After excluding patients without biopsy-proven PDAC, CCA, or GBCA and excluding patients without a CA 19-9 level at the time of diagnosis, 283 patients remained. Of these 283 patients, 23 (8.1%) had low, 70 (24.7%) had normal, and 190 (67.1%) had elevated CA 19-9 levels. The demographic characteristics of the study population stratified by CA 19-9 levels are shown in Table 2. Characteristics included age in years at the time of diagnosis, gender, BMI at time of diagnosis, race, and smoking history (never smoker, former smoker, or current smoker). Other characteristics abstracted from their medical charts included the history of other cancers, in addition to other medical comorbidities in individual patients including diabetes, hypertension, congestive heart failure, coronary artery disease, chronic obstructive pulmonary disease, and cerebrovascular accidents. The majority of patients across all three CA 19-9 groups were Hispanic (65.2% in the low group, 77.1% in the normal, and 54% in the elevated CA 19-9 groups), female (60.9% in the low, 57.1% in the normal, and 51.6% in the elevated CA 19-9 groups), and have never been smokers (82.6% in the low, 72.5% in the normal, and 60.5% in the elevated CA 19-9 groups). The median age was 60, 62, and 61 years in the low, normal, and elevated CA 19-9 level groups, respectively.

### 3.2. Oncologic Profile of the Study Population

The oncologic profile of the study population stratified by CA 19-9 level is shown in Table 3 with interquartile range values listed in parenthesis. CA 19-9 levels at the time of diagnosis ranged from 0 to 4,270,000 U/mL. The majority of patients across all three groups had a diagnosis of PDAC (60.9% in the low group, 55.7% in the normal group, and 69% in the elevated CA 19-9 group), an ECOG performance level of 0 (40.9% in the low group, 64.3% in the normal group, and 48.7% in the elevated CA 19-9 group), and stage IV disease (52.2% in the low group, 40% in the normal group, and 50.5% in the elevated CA 19-9 group) at the time of diagnosis. More than half of all patients with normal CA 19-9 levels (54.3%) underwent treatment with curative intent, while more than half of all patients with low and elevated CA 19-9 levels at 65.2% and 76.8% underwent palliative treatment. A total of 12 patients (52.2%) with low CA 19-9 levels, 28 patients (40%) with normal levels, and 96 patients (50.5%) with elevated levels had metastases at diagnosis.

### 3.3. Survival Analysis

**Figure 1:**
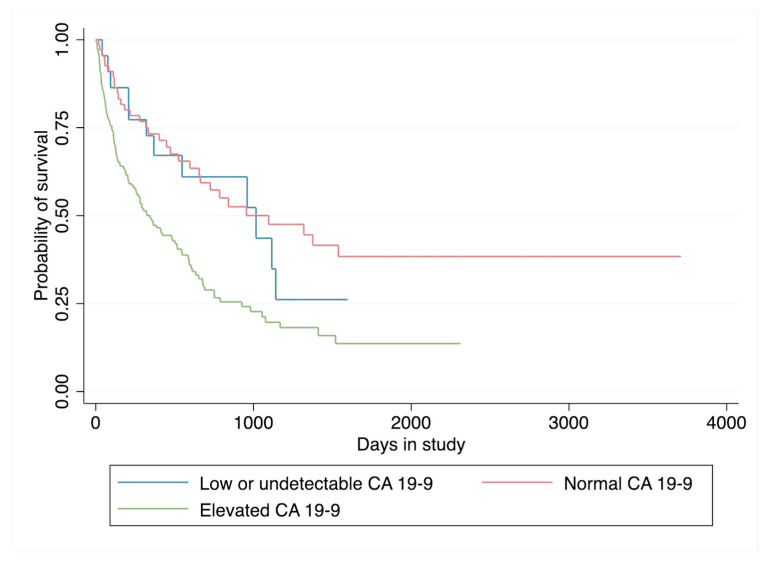
Kaplan–Meier estimates of survival in CA 19-9 groups.

Survival data and analyses are presented in Figure 1 and Table 3 and Table 4. A total of 164 patients (57.9%) died during a median follow-up period of 2.2 years. A total of 12 deaths (52.1%) occurred in the low group, 32 (45.7%) occurred in the normal group, and 120 (63.1%) occurred in the elevated group. After controlling for sex, age, BMI, the presence of metastases at the time of diagnosis, and treatment with curative intent, the hazard ratios for death in the normal and elevated CA 19-9 groups compared to the low group were 1.254 (*p* = 0.510, 95% CI 0.640–2.458) and 1.993 (*p* = 0.025, 95% CI 1.089–3.648), respectively (Table 4). The presence of metastasis at the time of diagnosis was associated with an increased hazard of death (HR = 1.815, *p* = 0.002, CI 1.252–2.629), while treatment with curative intent was associated with a decreased hazard of death (HR = 0.213, *p* < 0.001, CI 0.127–0.356).

## 4. Discussion

In this study, we examined the clinical features and outcomes of patients with tissue-proven PDAC, CCA, or GBCA and low, normal, or elevated CA 19-9 levels. Our study is the second to date that specifically examines the clinical features and outcomes of this patient population with PBMs who have low or undetectable CA 19-9 levels. We identified 286 LADHS patients with tissue-proven diagnoses of PBMs, 8.1% of whom had low CA 19-9 levels and 24.7% of whom had normal CA 19-9 levels at diagnosis. Given that PBMs are some of the most lethal malignancies due to late-stage presentation, early recurrence, and limited effective treatments, understanding trends in the clinical presentations and outcomes of patients with low CA 19-9 levels is important for their care [28,29].

CA 19-9 was initially described and adopted as a biomarker for hepatobiliary malignancies in 1979 by Koprowski et al., who used monoclonal antibodies to isolate antigens associated with colorectal carcinoma. It was subsequently found to also be associated with PDAC [19,30]. In 1983, Del Villano et al. developed a radioimmunometric assay to measure CA 19-9 levels that had high specificity and sensitivity for patients with PBMs [31]. Soon afterwards, CA 19-9 became a widely used and studied tumor marker for PBMs, particularly for diagnosis, prognosis, and monitoring response to treatment. Although CA 19-9 remains the most commonly used biomarker for PBMs, several limitations are known and should be considered when interpreting CA 19-9 levels clinically. In particular, CA 19-9 levels can be increased in other malignancies, including endometrial and ovarian cancers, and it can also be elevated in conditions such as benign biliary obstruction or chronic pancreatitis. Furthermore, some individuals are unable to produce CA 19-9 or produce CA 19-9 at lower levels, thus posing a challenge when interpreting CA 19-9 levels.

Across the literature, the CA 19-9 tumor marker has been studied for applications in predicting tumor stage and resectability, response to treatment, and overall survival. In terms of predicting the tumor stage, one study conducted at Massachusetts General Hospital evaluated how pre-operative CA 19-9 levels correlated with pathologic tumor stages. In 176 patients with pre- and postoperative CA 19-9 levels, preoperative CA 19-9 levels were found to be strongly associated with the stage of disease, with increased CA 19-9 levels in patients with higher stages of disease [32]. Similarly, CA 19-9 levels were lower in patients who had negative, compared to positive, lymph nodes. Additionally, several studies have examined CA 19-9 in association with disease resectability for PBMs. One study investigated the correlation between CA 19-9 levels and surgical resectability for PDAC; this study found that patients with resectable diseases had lower mean CA 19-9 levels compared to those with unresectable diseases [33]. A study examined preoperative CA 19-9 levels in 262 patients who underwent staging laparoscopy. This study found that preoperative CA 19-9 levels greater than or equal to 130 U/mL were a predictor of tumor unresectability on multivariate regression analysis (HR 2.70, *p* = 0.005) [34].

Furthermore, CA 19-9 levels have been studied for their applications in predicting response to treatment and monitoring a patient’s clinical course throughout therapy. Several studies have demonstrated that CA 19-9 levels before and after therapy can predict a patient’s response to the treatment and their overall survival. For example, in 1 study conducted on 43 patients with PDAC, the relationships between CA 19-9 levels before and after chemotherapy treatment, as well as the patients’ survival times, were investigated [35]. Patients who had a decrease in baseline CA 19-9 levels over 20% after 8 weeks of treatment had significantly better median survival times compared to those with an increase in CA 19-9 levels after treatment or those with a decrease of less than 20% of the baseline levels. In another study from Italy on patients with stage III or IV pancreatic adenocarcinoma, baseline CA 19-9 levels were found to correlate independently with survival. Moreover, patients with a decrease in CA 19-9 levels after chemotherapy of over 89% had a significantly improved median overall survival time compared to those with a decrease in CA 19-9 between 50 and 80% and those with a decrease of less than 50% or an increase in CA 19-9 levels [36].

Finally, CA 19-9 levels have been studied as a predictor of overall survival. A normal CA 19-9 level (defined as <37 U/mL) in patients with PBMs at the time of diagnosis has been reported as an independent predictor of survival [32,37,38]. In one Japanese study of 117 individuals with pancreatic carcinoma, patients with a normal CA 19-9 level had a 5-year disease-specific survival of 60% when compared to the 4% survival in patients with elevated CA 19-9 levels >37 U/mL [39]. In another study, overall survival was evaluated at 1, 3, and 5 years for patients with CA 19-9 less than or equal to 120 U/mL compared to CA 19-9 levels greater than 120 U/mL. Patients with CA 19-9 levels under 120 U/mL had significantly improved overall survival at 1, 3, and 5 years when compared to those with CA 19-9 levels greater than 120 U/mL (*p* = 0.002) [40]. Finally, decreased CA 19-9 levels after surgical resection in PDAC patients have been shown to predict survival. In one study, patients with a postresection CA 19-9 level above 90 U/mL had a significantly higher risk of death (HR 3.34; *p* <0.0001) when compared to those with levels less than or equal to 90 U/mL [41]. In a Japanese study of 269 patients, a postoperative CA 19-9 level above 37 U/mL was an independent predictor for poor survival (*p* < 0.0001) and was higher in patients with positive surgical margins (*p* = 0.02) [42].

The relationship between serum CA 19-9 levels and prognosis is confounded by the fact that 5–10% of the population have a Lewis-negative phenotype and do not produce the CA 19-9 tumor antigen despite having a PBM [11,43,44]. Because CA 19-9 is a sialyl Lewis antigen, patients without the sialyl Lewis phenotype are missing the enzyme 1,4-fucosyl transferase, which is required to produce this antigen. As a result, these patients may be mistaken to have negative or normal tumor marker levels, even when a metastatic PBM is present. 

In this study, we focused on the population of individuals with low or undetectable CA 19-9 levels in comparison with those with normal or elevated CA 19-9 levels. While we did not identify specific Lewis antigen phenotypes given the retrospective nature of this study, 8.1% of our patients had low CA 19-9 levels, which is similar to the proportion of patients with Lewis-negative phenotypes reported in the literature. In our largely Hispanic LADHS cohort, we found that the majority of patients in all three CA 19-9 groups presented with stage IV disease, which is consistent with the known late-stage presentation and morbidity of PBMs [26,27]. In addition, we found that patients with elevated CA 19-9 levels had a significantly increased hazard of death compared to patients with low CA 19-9, after controlling for sex, age, BMI, the presence of metastases, and treatment with curative intent. There was no significant difference in the hazard of death in patients with normal CA 19-9 compared to patients with low CA 19-9. These findings mirror those described by A.C. Berger et al., who studied 129 patients with pancreatic cancer from the Fox Chase Cancer Center database and found that the rates of survival in patients with low and normal CA 19-9 levels were similar to one another but significantly higher compared to those with elevated CA 19-9 levels [45]. In the context of a larger study with a greater sample of patients across several institutions, differences in survival between patients with low CA 19-9 levels compared to normal CA 19-9 levels can be more clearly distinguished.

A primary strength of this study is that this is one of the largest studies of low CA 19-9 level patients involving a predominantly Hispanic safety net population. While there are a multitude of studies that examine CA 19-9 levels in patients with pancreaticobiliary malignancies, the vast majority of these articles do not specifically identify patients with low or undetectable CA 19-9 levels. In addition, many of these studies were conducted on a vastly different patient population than the Hispanic-predominant LADHS population, including East Asian patients and European patients. According to the United States Census, the LADHS population has the largest Hispanic population of any county in the United States. Racial and ethnic minority patients continue to be vastly underrepresented in studies and clinical trials, and studies in pancreatic carcinoma have shown significant differences in incidence and overall survival among different ethnic groups [46]. This study, which highlights integral findings in an underrepresented population, fills a critical niche in the medical literature. Furthermore, this study not only comprehensively evaluated demographic data but also oncologic data for each group of patients, including CEA and CA 125 tumor markers, ECOG and tumor stage at the time of diagnosis, and the type of treatment the patients received. 

Several limitations should be acknowledged. First, this was a retrospective study that analyzed pre-existing data. Second, the study did not utilize a unified CA 19-9 lab assay, as both ADVIA Centaur and Elecsys E170 assays were employed in LADHS hospitals between 2014 and 2020. One assay defined an undetectable CA 19-9 level as <2 U/mL and the other as ≤3 U/mL; however, the threshold for normal CA 19-9 was 35 U/mL for both assays. Third, although the proportion of patients with low and undetectable CA 19-9 in our study (8.1%) is consistent with the reported proportion of patients with the Lewis antigen-negative phenotype (5–10%), specific Lewis antigen phenotypes were not determined for each of our study’s patients. 

## 5. Conclusions

Overall, few studies to date have focused on patients with low or undetectable CA 19-9 levels and particularly those with GBCA, CCA, and PDAC. This study adds to the small body of literature on patients with PBMs and low CA 19-9 levels, which is important given the morbidity and mortality of these malignancies. Our study showed that patients with low CA 19-9 levels have a significantly lower hazard of death compared to those with elevated CA 19-9 levels, despite our inability to monitor disease progression and response to treatment in this group of patients. Elevated CA 19-9 levels and the presence of metastases were associated with an increased hazard of death, while treatment with curative intent was associated with a decreased hazard of death. Additional multicenter studies are needed to continue investigating the significance of low or undetectable CA 19-9 levels in patients with PBMs and how best to care for this group of patients.

## Figures and Tables

**Table 1:** Below are 2021 ICD-10 codes for disease classification.

ICD-10 Code	Corresponding Disease Classification
C22.1	Intrahepatic bile duct carcinoma
C23	Malignant neoplasm of gallbladder
C25	Malignant neoplasm of pancreas
C25.0	Malignant neoplasm of head of pancreas
C25.1	Malignant neoplasm of the body of the pancreas
C25.2	Malignant neoplasm of tail of pancreas
C25.3	Malignant neoplasm of the pancreatic duct
C25.4	Malignant neoplasm of endocrine pancreas
C25.7	Malignant neoplasm of other parts of pancreas
C25.8	Malignant neoplasm of overlapping sites of pancreas
C25.9	Malignant neoplasm of pancreas, unspecified
D01.5	Carcinoma in situ of liver, gallbladder, and bile ducts

**Table 2:** Features of patients with low or undetectable serum CA 19-9 levels compared to those with normal and elevated CA 19-9 levels.

	Patients with Low CA 19-9 (n = 23)	Patients with Normal CA 19-9 (n = 70)	Patients with Elevated CA 19-9 (n = 190)
Age in years at diagnosis, median (IQR)	60 (52–69)	62 (53–70)	61 (55–66)
Male, n (%)	9 (39.1%)	30 (42.9%)	92 (48.4%)
BMI, median (IQR)	26.0 (23.2–30.6)	25.3 (22.4–28.7)	25.4 (22.2–29.9)
Race, n (%)			
Caucasian	3 (13.0%)	5 (7.1%)	31 (16.4%)
Hispanic	15 (65.2%)	54 (77.1%)	102 (54.0%)
Asian	2 (8.7%)	4 (5.7%)	21 (11.1%)
African American	2 (8.7%)	4 (5.7%)	15 (7.9%)
Middle Eastern/North African	0 (0.0%)	1 (1.4%)	2 (1.1%)
Other	1 (4.4%)	2 (2.9%)	18 (9.5%)
Comorbidities			
None	6 (26.1%)	23 (32.9%)	54 (28.4%)
Diabetes	9 (39.1%)	28 (40.0%)	78 (41.1%)
Hypertension	9 (39.1%)	31 (44.3%)	93 (49.0%)
Congestive heart failure	0 (0.0%)	3 (4.3%)	8 (4.2%)
Coronary artery disease	1 (4.4%)	5 (7.1%)	11 (5.8%)
COPD	0 (0.0%)	0 (0.0%)	8 (4.2%)
Cerebrovascular accident	0 (0.0%)	1 (1.4%)	8 (4.2%)
History of other cancer, n (%)			
None	21 (91.3%)	61 (88.4%)	175 (92.1%)
Non-GI	2 (8.7%)	5 (7.2%)	12 (6.3%)
GI	0 (0.0%)	4 (5.8%)	3 (1.6%)
Smoking history, n (%)			
Never	19 (82.6%)	50 (72.5%)	113 (60.1%)
Former	1 (4.4%)	14 (20.3%)	47 (25.0%)
Current	3 (13.0%)	5 (7.3%)	28 (14.9%)

*p*-values omitted as there were no significant differences between groups. Abbreviations: BMI: Body mass index; GI: gastrointestinal; IQR: interquartile range.

**Table 3:** Oncologic data for patients with low or undetectable serum CA 19-9 levels compared to those with detectable CA 19-9 levels.

	Patients with Low CA 19-9 (n = 23)	Patients with Detectable but Normal CA 19-9 (n = 70)	Patients with Elevated CA 19-9 (n = 190)	*p*-Value
CA 19-9 level at diagnosis, median (IQR)	2.0 (2.0–3.0)	16.5 (9.0–22.0)	532.5 (147.0–3469.0)	0.595
CEA level at diagnosis, median (IQR)	4.9 (2.8–6.6)	2.0 (1.4–5.1)	4.1 (1.9–17.8)	0.508
CA 125 level at diagnosis, median (IQR)	30.6 (6.1–55.0)	26.7 (10.4–48.9)	61.3 (20.9–219.0)	0.646
Total bilirubin at diagnosis, median (IQR)	0.6 (0.5–4.0)	1.0 (0.6–2.1)	1.7 (0.8–8.6)	0.002
Organ system with malignancy, n (%)				0.054
Cholangiocarcinoma	5 (21.7%)	18 (25.7%)	46 (24.2%)	
Gallbladder adenocarcinoma	4 (17.4%)	13 (18.6%)	13 (6.8%)	
Pancreatic adenocarcinoma	14 (60.9%)	39 (55.7%)	131 (69.0%)	
Deceased by end of study period, n (%)	12 (52.2%)	32 (45.7%)	120 (63.2%)	0.035
Survival time, median *	1016	1096	344	-
Follow-up time, median **	815	1243	662	-
ECOG at time of diagnosis, n (%)				0.236
0	9 (40.9%)	45 (64.3%)	90 (48.7%)	
1	7 (31.8%)	17 (24.3%)	54 (29.2%)	
2	4 (18.2%)	5 (7.1%)	17 (9.2%)	
3	2 (9.1%)	2 (2.9%)	22 (11.9%)	
4	0 (0.0%)	1 (1.4%)	2 (1.1%)	
Stage at time of diagnosis, n (%)				0.017
I	4 (17.4%)	19 (27.1%)	21 (11.1%)	
II	4 (17.4%)	17 (24.3%)	34 (17.9%)	
III	3 (13.0%)	6 (8.6%)	39 (20.5%)	
IV	12 (52.2%)	28 (40.0%)	96 (50.5%)	
Underwent curative treatment, n (%)	8 (34.8%)	38 (54.3%)	44 (23.2%)	<0.001
Type of treatment, n (%)				<0.001
Chemotherapy	12 (52.2%)	20 (28.6%)	92 (48.4%)	
Surgery	3 (13.0%)	16 (22.9%)	14 (7.4%)	
Chemotherapy and surgery	5 (21.7%)	26 (37.1%)	36 (19.0%)	
Hospice	3 (13.0%)	6 (8.6%)	43 (22.6%)	
None or lost to follow-up	0 (0.0%)	2 (2.9%)	5 (2.6%)	

* Obtained from the Kaplan–Meier estimate of the survivor function. ** Obtained using the reverse Kaplan–Meier estimator. Abbreviations: CEA: Carcinoembryonic antigen; CA 125: cancer antigen 125; ECOG: Eastern Cooperative Oncology Group performance status; IQR: interquartile range.

**Table 4:** Cox proportional hazards regression modeling of survival.

Predictor	Hazard Ratio	*p*-Value	95% Confidence Interval
CA 19-9 level			
Undetectable (reference)	-	-	-
Normal	1.254	0.510	0.640–2.458
Elevated	1.993	0.025 *	1.089–3.648
Male	0.973	0.866	0.709–1.336
Age	1.016	0.052	1.000–1.033
BMI	1.001	0.923	0.977–1.026
Evidence of metastases at time of diagnosis	1.815	0.002 *	1.252–2.629
Underwent curative treatment	0.213	<0.001 *	0.127–0.356

* Denotes statistical significance.

## Data Availability

The data presented in this article are available on request by the corresponding author.
